# Demographic determinants of acute gastrointestinal illness in Canada: a population study

**DOI:** 10.1186/1471-2458-7-162

**Published:** 2007-07-18

**Authors:** Shannon E Majowicz, Julie Horrocks, Kathryn Bocking

**Affiliations:** 1 Department of Population Medicine, University of Guelph, Guelph, Ontario, Canada; 2 Foodborne, Waterborne and Zoonotic Infections Division, Public Health Agency of Canada, Guelph, Ontario, Canada; 3 Department of Mathematics and Statistics, University of Guelph, Guelph, Ontario, Canada

## Abstract

**Background:**

Gastrointestinal illness is an important global public health issue, even in developed countries, where the morbidity and economic impact are significant. Our objective was to evaluate the demographic determinants of acute gastrointestinal illness in Canadians.

**Methods:**

We used data from two population-based studies conducted in select communities between 2001 and 2003. Together, the studies comprised 8,108 randomly selected respondents; proxies were used for all respondents under 12 years and for respondents under 19 years at the discretion of the parent or guardian. Using univariate and multivariate logistic regression, we evaluated the following demographic determinants: age, gender, cultural group, and urban/rural status of the respondent, highest education level of the respondent or proxy, number of people in the household, and total annual household income. Two-way interaction terms were included in the multivariate analyses. The final multivariate model included income, age, gender, and the interaction between income and gender.

**Results:**

After adjusting for income, gender, and their interaction, children under 10 years had the highest risk of acute gastrointestinal illness, followed by young adults aged 20 to 24 years. For males, the risk of acute gastrointestinal illness was similar across all income levels, but for females the risk was much higher in the lowest income category. Specifically, in those with total annual household incomes of less than $20,000, the odds of acute gastrointestinal illness were 2.46 times higher in females than in males.

**Conclusion:**

Understanding the demographic determinants of acute gastrointestinal illness is essential in order to identify vulnerable groups to which intervention and prevention efforts can be targeted.

## Background

Gastrointestinal illness (GI) remains an important global public health issue [1,2]. In developed countries, although GI tends to be self-limiting and mild, the associated morbidity and economic impact are significant [3-5]. To address this, numerous countries have estimated the incidence and burden of GI in the community via population-based studies [6-12], including two studies conducted recently in Canadian communities [13,14]. These population-based studies collect information on gastrointestinal symptoms experienced by a random sample of study area residents, the severity of those symptoms, and demographic and other information. Since the main purpose of these studies is to estimate disease incidence and burden, demographic determinants of illness are often investigated as a secondary objective only, and are not necessarily thoroughly explored.

Understanding the relationships between GI and determinants of health in the general population, however, is essential to identify vulnerable groups to which intervention and prevention efforts can be targeted. Therefore, the objective of this study was to investigate the demographic determinants of acute gastrointestinal illness (AGI) in Canadians using available data from population-based studies.

## Methods

### Data sources

At the time of this analysis, the Public Health Agency of Canada (formerly Health Canada) had conducted two studies designed to ascertain the burden and distribution of self-reported AGI in defined Canadian populations. One study was conducted in Hamilton, Ontario, Canada from February 2001 to February 2002, and one was conducted in three communities in the province of British Columbia from June 2002 to June 2003; these studies used the same methodology and core survey tool, and have been described in detail elsewhere [13,14]. Briefly, both were retrospective, cross-sectional telephone surveys administered to randomly selected residents of the study area. A random sample of households was chosen using a commercial database of residential telephone numbers. One individual from each household was then randomly selected to participate by identifying the individual in the household with the next birthday. Proxy respondents were used for all individuals under 12 years of age, and for individuals 12 to 18 years of age at the discretion of the parent or guardian.

Respondents were asked whether they had experienced any vomiting or diarrhea in the 28 days prior to the interview. Cases were those respondents who reported vomiting or diarrhea in the four weeks prior to the interview, excluding those who reported that their vomiting or diarrhea was due to a chronic condition including pregnancy, medication use, colitis, diverticulitis, Crohn's disease, irritable bowel syndrome, or other chronic condition. Respondents who did not report vomiting or diarrhea, as well as those whose symptoms were due to chronic conditions, were included in the non-case group. A broad case definition for AGI was deliberately chosen to ensure high sensitivity and case capture.

Ethical approval for these studies was obtained from one or more of the following boards: the Research Ethics Board of St. Joseph's Hospital (Hamilton, Ontario, Canada), McMaster University (Hamilton, Ontario, Canada), the Human Subjects Committee of the University of Guelph (Guelph, Ontario, Canada), and the University of British Columbia Behavioural Research Ethics Board (Vancouver, British Columbia, Canada). The response rates for the surveys were 36.6% [13] and 44.3% [14], which are in the range of response rates for similar studies [7,9,15,16]. Together, the studies comprised 8,108 respondents (Hamilton, 3,496; British Columbia, 4,612).

### Statistical methods

The demographic determinants of illness and possible confounding factors included in this analysis are listed in Table 1. Age, gender and culture were individual-level variables referring to characteristics of the randomly selected respondent, while urban/rural status and income were household level variables. Final age categories were determined by grouping together five-year age categories with similar risks of AGI. Culture was defined as the cultural group with which the respondent most identified. Education was defined as the highest level attained by the respondent, or by the proxy for those respondents 18 years and younger. This was done in an attempt to capture the education of the person guiding the behaviour of the respondent. Income was defined as the total gross annual household income. Urban/rural status was defined using the Statistics Canada classification scheme in which urban areas are those with a minimum population of 1000 persons and a population density of at least 400 persons per square kilometer, and all other areas are rural. Respondents were assigned urban/rural status by linking their reported residential postal code to the corresponding Statistics Canada classification using a commercial database (Enhanced Postal Code Conversion File, Desktop Mapping Technologies, Inc., Markham, Ontario, Canada).

**Table 1:** Frequency (n), percent (%), odds ratios (OR), 95% confidence intervals (CI) for odds ratios and *P*-values from the univariate analysis of the relationship between demographic determinants and acute gastrointestinal illness in randomly selected residents of two Canadian study areas, 2001 to 2003 (N = 8,108)

Variable	n	%	OR	CI for OR	*P*-value
Number of People in Household					0.005 ^a^
One	2163	26.68	0.76	(0.63, 0.91)	0.004
Two	2644	32.61	0.80	(0.68, 0.95)	0.012
Three or more ^b^	3220	39.71	1.00	-	-
Missing	81	1.00			
Urban/Rural Status					0.080 ^a^
Urban ^b^	5957	73.47	1.00	-	-
Rural	1486	18.33	0.84	(0.69, 1.02)	0.080
Missing	751	9.31			
Education					0.150 ^a^
No high school diploma ^b^	1323	16.32	1.00	-	-
High school diploma	3190	39.34	1.20	(0.96, 1.51)	0.124
College/trade school diploma	1271	15.66	1.30	(1.00, 1.70)	0.052
University, graduate, or professional diploma	1648	20.33	1.31	(1.02, 1.69)	0.035
Missing	601	8.35			
Culture					<0.001 ^a^
North American ^b^	6492	80.07	1.00	-	-
European	762	9.40	0.75	(0.57, 0.98)	0.038
African	67	0.83	0.26	(0.04, 0.83)	0.061
Mediterranean	88	1.09	0.62	(0.24, 1.31)	0.259
Asian	427	5.27	0.37	(0.22, 0.58)	<0.001
Native North American/Aboriginal	91	1.12	1.54	(0.83, 2.65)	0.142
South American	31	0.38	0.91	(0.22, 2.57)	0.872
Austral-Asian	24	0.30	0.77	(0.12, 2.62)	0.723
Missing	126	1.55			
Income					0.033 ^a^
<$20 000^b^	951	11.73	1.00	-	-
>$20 000 to <$40 000	1415	17.45	0.78	(0.61, 1.02)	0.074
>$40 000 to <$60 000	1469	18.12	0.77	(0.59, 1.00)	0.047
>$60 000 to <$80 000	987	12.17	1.07	(0.82, 1.40)	0.662
>$80 000	1188	14.65	0.82	(0.62, 1.07)	0.138
Missing	2098	25.88			
Age (years)					<0.001 ^a^
0–9 ^b^	562	6.93	1.64	(1.29, 2.09)	<0.001
10–14	323	3.98	1.12	(0.78, 1.57)	0.527
15–19	319	3.93	0.90	(0.61, 1.30)	0.605
20–24	339	4.18	1.59	(1.16, 2.14)	0.003
25–64	4747	58.55	1.00	-	-
65–69	448	5.53	0.60	(0.40, 0.87)	0.009
70–74	387	4.77	0.32	(0.18, 0.52)	<0.001
75–84	413	5.09	0.43	(0.26, 0.66)	<0.001
>84	98	1.21	0.36	(0.11, 0.86)	0.045
Missing	472	5.82			
Gender					0.001 ^a^
Male ^b^	3254	40.13	1.00	-	-
Female	4828	59.55	1.28	(1.11, 1.50)	0.001
Missing	26	0.32			

^a ^Global *P*-value for Wald test of H_o_: same level of risk in all categories

^b ^reference group

Logistic regression was used to determine how the risk of AGI related to demographic variables. To examine whether the relationship between each demographic factor and the risk of AGI varied between the two study areas, we fit (separately for each demographic factor) multivariate models with the demographic factor, study area, and their interaction as independent variables (results not shown). However, no significant interactions were found and data from the two studies were combined for all further analyses. Univariate models were then fit for each demographic variable (Table 1). To explore various multivariate models and ultimately choose a final model, all demographic variables and their two-way interactions were used in stepwise selection, with the criteria for entry and exit into the model being that the *P*-value of the score chi-square test be less than 0.05. Individuals with missing data for a given variable were excluded from any models in which that variable was present. To assess whether any variables in the final model were subject to confounding by any variables that had been omitted from the final model, each omitted variable was re-introduced individually (results not shown). The impact on the sign, magnitude, and significance of each of the original coefficients was examined; a change from significant to non-significant (or vice versa) at *P *= 0.05, or a change in the resulting odds ratio of ± 0.5, was considered biologically significant enough to retain the variable in the final model as a confounder.

All statistical analyses were carried out in SAS version 9.1 (SAS Institute Inc., Cary, NC, USA, 2002–2003). Likelihood ratios were used to compare models and Wald tests were used for tests of global hypotheses and tests involving individual parameters [17].

## Results

### Univariate analysis

Results of the univariate analysis are shown (Table 1). The risk of AGI was significantly associated with the number of people in the household (*P *= 0.005), culture (*P *=< 0.001), income (*P *= 0.033), age (*P *< 0.001), and gender (*P *= 0.001), but not with urban/rural status (*P *= 0.080) or education (*P *= 0.150). Specifically, the risk of AGI in the past four weeks increased significantly as the number of people in the household increased.

The odds of AGI for respondents who identified themselves as Asian were only 0.37 times higher (i.e. 2.69 times lower) than respondents who identified themselves as North American (*P *< 0.001). Respondents with household incomes between $40,000 and $60,000 had odds of AGI that were 0.76 times higher (i.e. 1.32 times lower) than respondents with household incomes less than $20,000 (P = 0.047). A significantly higher risk of AGI was observed in children less than 10 years (*P *< 0.001), and young adults 20 to 24 years (*P *= 0.003), compared to those 25 to 64 years. The odds of AGI in females were 1.28 times higher than males (*P *= 0.001).

### Multivariate analysis

The final multivariate model included income, age, gender, and the interaction between income and gender. Re-introduction of the variables excluded from the final model (number of people in the household, urban/rural status, education, and culture) did not impact the sign, magnitude, or significance of any of the coefficients for income, age, gender, or the interaction between income and gender. Therefore, income, age, gender, and the income-gender interaction were the variables included in the final multivariate model (Table 2).

**Table 2:** Adjusted odds ratios (OR), 95% confidence interval (CI) for odds ratios and *P*-values from the final multivariate model of the relationship between demographic determinants and acute gastrointestinal illness in randomly selected residents of two Canadian study areas, 2001 to 2003 (N = 5,732)

Variable	OR	CI for OR	*P*-value
Intercept	0.15	(0.10, 0.22)	<0.001
Income			0.819 ^a^
<$20 000^b^	1.00	-	-
>$20 000 to <$40 000	1.23	(0.75, 2.07)	0.390
>$40 000 to <$60 000	1.06	(0.64, 1.74)	0.827
>$60 000 to <$80 000	1.26	(0.75, 2.09)	0.382
>$80 000	1.21	(0.73, 1.99)	0.451
Age (years)			<0.001 ^a^
0–9	1.50	(1.14, 1.96)	0.003
10–14	1.23	(0.84, 1.79)	0.290
15–19	0.85	(0.52, 1.38)	0.510
20–24	1.48	(1.03, 2.14)	0.035
25–64 ^b^	1.00	-	-
65–69	0.51	(0.33, 0.80)	0.003
70–74	0.30	(0.16, 0.56)	<0.001
75–84	0.36	(0.21, 0.61)	<0.001
>84	0.27	(0.08, 0.89)	0.031
Gender			<0.001^a^
Male ^b^	1.00	-	-
Female	2.46	(1.54, 3.93)	<0.001
Income*Gender Interaction			0.017 ^a^
>$20 000 to <$40 000 (Female)	0.46	(0.25, 0.84)	0.011
>$40 000 to <$60 000 (Female)	0.50	(0.28, 0.91)	0.022
>$60 000 to <$80 000 (Female)	0.56	(0.30, 1.02)	0.059
>$80 000 (Female)	0.35	(0.19, 0.64)	<0.001

^a ^Global *P*-value for Wald test of H_o_: same level of risk in all categories

^b ^reference group

**Figure 1:**
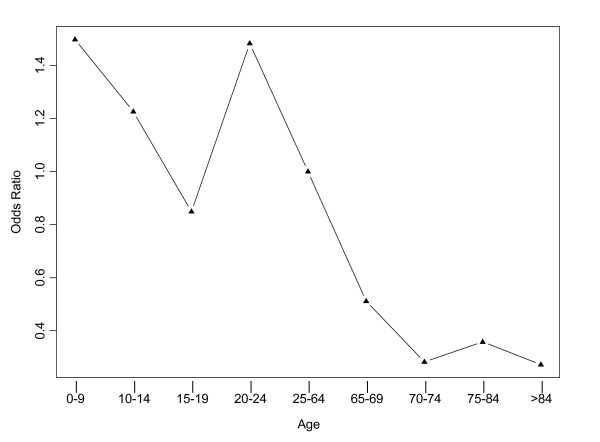
Adjusted odds ratios for the risk of acute gastrointestinal illness by age (in years), adjusted for income, gender, and their interaction, in randomly selected residents of two Canadian study areas, 2001 to 2003 (N = 5,732). Reference age group is 25–64 years.

The results of the multivariate analysis and univariate analysis were consistent with respect to age. Odds ratios by age group, adjusted for income, gender, and their interaction, are shown (Figure 1). Even after adjusting for income, gender, and the interaction between income and gender, children under 10 had the highest risk of AGI, followed by young adults aged 20 to 24. In the multivariate model, and Figure 1, the reference age group is 25–64 years, so the adjusted odds ratio is 1 for this age group. The adjusted odds ratios range from 0.27 in those older than 84 years, to 1.50 in those under 10 years. Thus, for instance, the odds of AGI in 0–9 year olds was 1.50 times that of 25–64 year olds, adjusting for income and gender.

**Figure 2:**
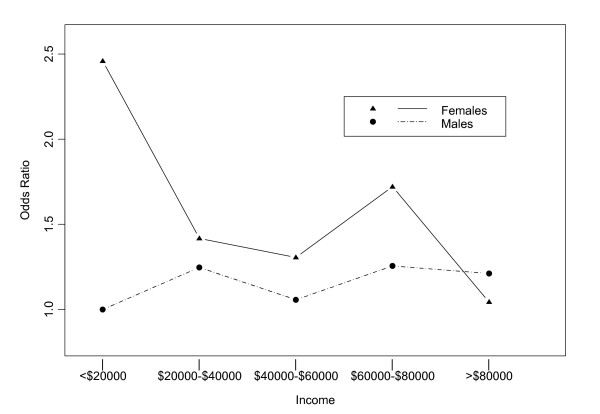
Change in risk of acute gastrointestinal illness across income levels for males (dashed line) versus females (solid line), adjusted for age. Age-adjusted odds ratios are shown on the y axis, total annual household income is shown on the x axis (N = 5,732). Reference group is males with incomes less than $20,000.

We found a significant interaction between income and gender. Figure 2 shows the odds ratios for different gender/income groups, compared with the reference group of males with incomes less than $20,000, adjusted for age. For instance, males earning $60,000 – $80,000 had an odds of AGI that was 1.26 times that of males earning less than $20,000, adjusting for age-group. On the other hand, females earning $60,000 – $80,000 had an adjusted odds of AGI that was 1.74 times that of males earning less than $20,000. (This number can be found by multiplying together the odds ratios for income of $60,000–$80,000, females, and their interaction: 1.26 *2.46*0.56 = 1.74).

It is interesting to compare the odds of AGI in males and females with the same income. For instance, the odds ratio for females earning $60,000–$80,000 versus males with the same income was 1.38 (1.74/1.26). This difference is not significant (P = 0.107). The difference between the odds of AGI in males and females was statistically significant only at the lowest income level (*P *< 0.001). In those with total annual household incomes of less than $20,000, the odds of AGI were 2.46 times higher in females than in males.

In Figure 2, the differential effect of income on the risk of AGI for males and females is clearly visible. For males, the risk of AGI was approximately constant across all income levels. For females, however, the risk was much higher in those with total household incomes less than $20,000.

## Discussion

This study investigated the demographic determinants of AGI in select Canadian-based populations. Adjusting for gender and income, children under 10 years had the highest risk of AGI, followed by those aged 20 to 24 years. Adjusting for age, in households with annual incomes under $20,000, females were significantly more likely to have AGI than males. These results highlight that, although AGI is typically self-limiting and is rarely a cause of mortality in developed countries, it still represents a particular health risk in specific sub-populations.

Interestingly, there was no interaction between the place in which the study occurred and each of the demographic variables assessed. Thus, it is possible that the associations observed here are consistent across two distinct geographic areas of Canada (southern Ontario, British Columbia), such that the increased risk in low income females and children may be due to underlying factors unrelated to geography. Additionally, income, age, and gender were significantly associated with the risk of AGI whether or not the following variables were controlled for: total number of people in the household, the urban/rural status of the respondent, education, and cultural group, suggesting that the observed associations are not confounded by these variables.

In the univariate analysis, we observed a significantly higher risk of AGI in children under 10 years, and young adults between 20 and 24 years, as compared to adults aged 25 to 64 years. As noted above, even when income, gender, and the income-gender interaction were accounted for, children under 10 years and young adults between 20 and 24 years remained at a higher risk of AGI. In children, this increased risk likely reflects an increased susceptibility due to immune status. In young adults, this increased risk may reflect behavioural factors. Our findings are somewhat consistent with one study from the Netherlands, which reported highest incidences in children and the elderly [6], and with other international studies which report higher rates in children [8,9,18,19]. Interestingly, we did not observe an increased risk of AGI in the elderly, as has been observed elsewhere [6] and which is biologically plausible due to decreasing immune function. Although individuals over the age of 65 years were well represented in our data (n = 1,346), it is likely that the sampling method inadvertently selected for healthier individuals in this age category, since residents of institutions (including nursing homes) were not included in the sampling frame. Thus, despite the lack of observed risk in the elderly in this study, it is possible that elderly Canadians may be at increased risk for AGI.

Past studies report higher rates of AGI in females than males [6,13,14,20,21], which may be due to an increased exposure to infectious causes of AGI via foodborne or person-to-person transmission. Given that the kitchen serves as a reservoir for many foodborne causes of AGI [22], the higher risk observed in females may reflect the fact that, in Canada, females generally do more food preparation than males and thereby incur greater exposure to pathogens. This is supported by a study from England and Wales which found female gender a significant risk for infection with *Campylobacter jejuni *infection [23], which is primarily a foodborne pathogen [24]. Additionally, although the relationship between gender and the risk of AGI observed here was unconfounded by the number of people in the household, we were unable to account for the ages of any household residents other than the respondent. In a recent Australian study [21], women between the ages of 25 and 64 years both with and without AGI were compared; the study found that 18% of women with AGI had at least one child under five years of age in their household, compared to 5% of women without AGI. Thus, it is possible that females are at an increased risk due to the presence of and interaction with young children within the household.

It is possible that the higher rate of AGI observed in females may be due to recall bias (where females may be more likely to take note of and thus report AGI than males), or to reporting bias (where females may be more comfortable discussing and thus more likely to report AGI than males). However, such explanations are unlikely given the plausibility for greater exposure to infectious causes of AGI in females. In addition, if reporting bias was the reason for the higher rate of AGI observed in females, we would expect higher rates in females consistently across age groups and income levels, which was not observed here.

There is a growing body of literature linking health to income or income inequality, with lower household income associated with an increased risk of morbidity and mortality [25-28]. In Canada, income and good health are positively associated [26,29], and low socio-economic status is associated with poor health [30], despite the existence of universally insured health services. However, the specific association between income and the risk of AGI has not been previously explored.

Here, we found that total annual household income was associated with the risk of AGI in females, but not males. In males, the risk of AGI was consistent regardless of income. In females, a higher risk occurred in those in the lowest income category (total annual household income of less than $20,000). In this category, the odds of AGI for females were 2.5 times higher than the odds for males, regardless of age. Unfortunately, no literature exists which provides explicit reasons for this observation. Specific hypotheses may include different occupational risk settings in low income males versus females, or an increased susceptibility in females (due perhaps to increased foodborne exposure or increased exposure to infected children) that is exacerbated by low income living conditions. Further research evaluating reasons for this apparent increased risk in low-income females is warranted. Regardless of the cause, this finding calls for targeting information and interventions to this segment of the population, potentially via local public health outreach programs.

The results of the multivariate analysis can be interpreted to yield stratum-specific odds ratios; for example, the odds of AGI in a female child under 10 years of age who resides in a low income household (i.e. total annual household income of less than $20,000) is 0.554, and the odds of AGI in a male child in the same age and income category is 0.225, yielding an odds ratio of 2.46. Examining the risk of AGI in females across income categories showed that those in households with total annual incomes of $20,000 to $40,000 (OR = 0.566), $40,000 to $60,000 (OR = 0.530), $60,000 to $80,000 (OR = 0.706), and over $80,000 (OR = 0.424) all had a lower risk of AGI than females in low income households.

Low response rate in the original studies (36.6% in Hamilton and 44.3% in B.C) was the main limitation of this study, and is a limitation typical of such telephone surveys. Other similar studies report response rates ranging from 27% to 71% [6,7,9,15,16] Non-response in our data likely related to the subject-matter of the questionnaire. If the nature of the relationship between AGI and demographic determinants in respondents is different than in the non-respondents, then the results presented here will be biased. Future studies should attempt to minimize non-response to mitigate this potential source of bias.

Item non-response is also a concern in surveys. Here, urban/rural status was missing for 9% of respondents, and total annual household income was missing for 25% of respondents. Thus our final multivariate model, which included income, used data from 5,732 respondents. This may be of concern if the likelihood of responding to specific questions differed across income levels. For example, if non-response to the income question is greater in those with lower incomes, and low income is a risk factor for AGI, the odds ratio for low versus high income will be a biased underestimate of the true risk of illness. To address this, we also examined models without income (results not shown). Since such analyses yielded consistent conclusions with respect to the other demographic variables, the impact of income non-response on the results presented here is likely minor.

In our study, the education variable measured the highest level of education attained by either the respondent (for those over 18 years) or their proxy (for those 18 years and younger), in an attempt to capture the education of the person guiding the behaviour of the respondent. Thus, for those 18 years and under, we assumed that the proxy (parent or legal guardian) was more instrumental in guiding the behaviour of the respondent than the respondent themselves. Although this is likely true for very young respondents, it may be less true as the age of the respondent approaches 18 years. Thus for teenage respondents, we may have over-estimated the education level of the person guiding the behaviour of the respondent. However, in our analysis, we found no interaction between education and age, suggesting that the lack of association between education and the risk of AGI observed here is the same regardless of the age of the respondent. In any case, our findings do not negate the need for future in-depth analyses of specific components of education (e.g. food handling or hygiene training) that may decrease the risk of AGI.

Cases of AGI in this analysis were those who reported vomiting or diarrhea in the past four weeks, excluding those whose symptoms were due to a chronic condition. In these data, no attempt was made to differentiate infectious AGI from other causes such as food allergies or intolerances, over-indulgence of drugs or alcohol, or other causes of AGI. Although respondents reported what they believed to be the cause of their illness, this information was not used to exclude cases since the validity of these self-diagnosed causes was highly variable. Thus our case definition of AGI, although highly sensitive for infectious GI (i.e. includes most true cases of infectious GI), should not be considered specific for infectious GI (i.e. includes some non-infectious cases of AGI).

Another possible limitation of this study is that the data were collected via telephone interview. Thus, the results presented here may not be applicable to those without telephones, such as the homeless, those in institutions, or who are incarcerated. Lastly, it is debated whether analyses such as these should be weighted by the number of persons in the household [31]. We repeated our analyses (not shown) with weighting and found no difference in the substantive conclusions.

## Conclusion

In Canada, it appears that children less than 10 years, young adults 20 to 24 years, and females in households with annual incomes under $20,000 are at an increased risk for AGI. In children, this increased risk may reflect an increased susceptibility to gastrointestinal infections due to immune status, and in young adults, this increased risk may be due to behavioural factors. In low income females, however, the specific reasons for this increased risk are unclear, and further research is needed. Understanding these relationships between AGI and determinants of health in the population is necessary to guide intervention and prevention efforts. These results suggest that children, young adults, and low income females should be targeted by public health programs aimed at decreasing the incidence of AGI in Canada.

## Competing interests

The author(s) declare that they have no competing interests.

## Authors' contributions

SM and JH planned the analysis of the data. KB performed the statistical analysis. All authors were involved in interpreting the results and writing the manuscript. All authors have read and approved the final manuscript.

## Pre-publication history

The pre-publication history for this paper can be accessed here:


